# Development of a UPLC-MS/MS method for the determination of lacosamide and its metabolite and its application to drug-drug interaction

**DOI:** 10.3389/fphar.2023.1265252

**Published:** 2023-11-10

**Authors:** Jie Chen, Yuxin Shen, Hailun Xia, Xiaohai Chen, Ren-Ai Xu, Guanyang Lin, Gexin Dai

**Affiliations:** ^1^ Department of Pharmacy, The First Affiliated Hospital of Wenzhou Medical University, Wenzhou, Zhejiang, China; ^2^ School of Pharmaceutical Sciences, Wenzhou Medical University, Wenzhou, Zhejiang, China; ^3^ Key Laboratory of Diagnosis and Treatment of Severe Hepato-Pancreatic Diseases of Zhejiang Province, The First Affiliated Hospital of Wenzhou Medical University, Wenzhou, Zhejiang, China

**Keywords:** methodological verification, lacosamide, UPLC-MS/MS, interaction, pharmacokinetic

## Abstract

Lacosamide, a third-generation novel antiepileptic drug, was first approved in 2008 as an adjunct to partial seizures. In 2014, the U.S. Food and Drug Administration (FDA) approved it as a single agent for partial seizures. Since epilepsy is a chronic condition, most patients need long-term antiepileptic medicinal products, so it is even more important to consider the drug-drug interactions (DDIs). For the purpose of this experiment, an ultra performance liquid chromatography tandem mass spectrometry (UPLC-MS/MS) assay with accuracy and simplicity was optimized and fully validated for the simultaneous quantitative determination of lacosamide and O-Desmethyl-lacosamide (ODL), and DDIs between lacosamide and nisoldipine *in vivo* and *in vitro* was researched. The protein was precipitated with acetonitrile, the analytes were eluted with acetonitrile and a 0.1% formic acid solution in a gradient program, and lacosamide, ODL, and lamotrigine (Internal Standard, IS) were successfully separated by chromatography. The findings of the biological analysis revealed that the lower limit of quantification (LLOQ) for lacosamide in samples was 2 ng/mL and the linearity ranged from 2 to 10000 ng/mL. The LLOQ for ODL was 1 ng/mL, while the linearity range for this substance was 1–1,000 ng/mL. In rat liver microsomes (RLM), the LLOQ of ODL was 80 ng/mL and the linear range was 80–40000 ng/mL. The selectivity, stability, matrix effect and recovery rate were all satisfied with the need of quantitative analysis of samples. Then, the UPLC-MS/MS assay was employed successfully on the interactions of lacosamide and nisoldipine *in vivo* and *in vitro*. The half-maximal inhibitory concentration (IC_50_) was 3.412 μM in RLM, where nisoldipine inhibited the metabolism of lacosamide with a mixture of inhibition mechanism. In rat pharmacokinetic experiments, it was found that nisoldipine could significantly change the pharmacokinetic characteristics of lacosamide, including AUC_(0-t)_, AUC_(0-∞)_, T_max_, CL_z/F_ and C_max_, but had no significant effect on ODL. In summary, the UPLC-MS/MS method could accurately and sensitively quantify lacosamide and ODL, and could be used for the interaction between nisoldipine and lacosamide *in vivo* and *in vitro*.

## Introduction

Approximately 70 million individuals globally are suffering from epilepsy, among the most prevalent brain disorders. It is distinguished by a propensity to repeatedly cause spontaneous seizures, which have a variety of neurobiological, cognitive, and psychosocial effects (Thijs et al., 2019). The first generation of antiepileptic drugs had more adverse reactions, mainly in cognitive aspects, and were rarely used in clinical practice (Nanau and Neuman, 2013; Steinhoff and Staack, 2019). The second generation of antiepileptic drugs had relatively fewer side effects than the first generation and were slightly better at controlling seizures (Perucca et al., 2020).

**FIGURE 1:**
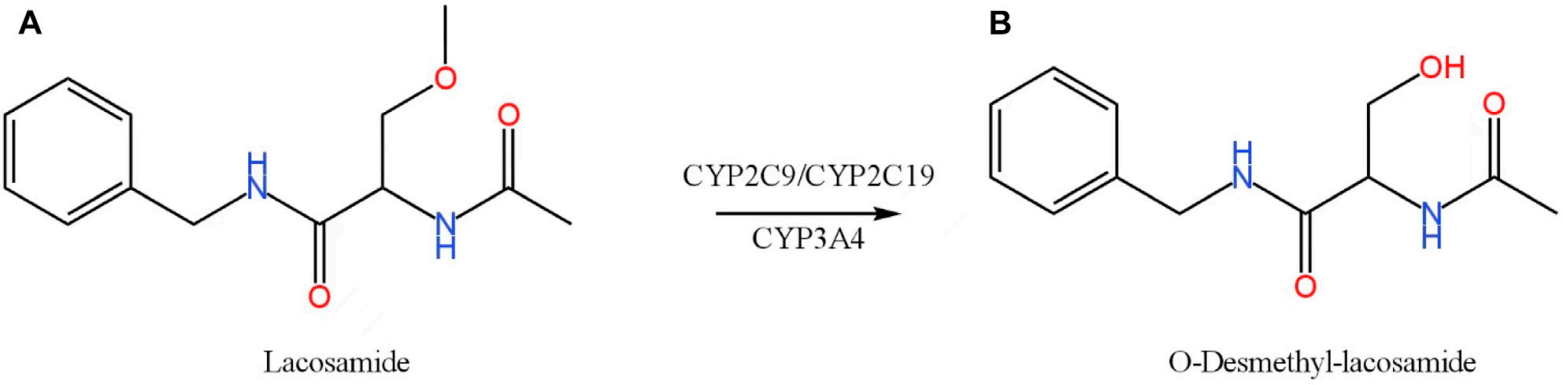
Chemical structures and biotransformation pathway of lacosamide **(A)** and O-Desmethyl-lacosamide **(B)**.

Lacosamide (Figure 1A), as a third-generation novel antiepileptic drug, stands out for its good efficacy and safety, with better pharmacokinetic characteristics and tolerability compared to the first and second generation (Carona et al., 2021). It is used adjunctively for the treatment of adults with or without secondary systemic partial seizures, with a low incidence of adverse effects, and is now available for the treatment of primary generalized tonic-clonic seizures or generalized epilepsy, in addition to being shown to be therapeutic as adjunctive therapy for focal epilepsy (Kim et al., 2012; Ahn et al., 2022).

Antiepileptic drugs often require other drugs in combination with multiple therapies to control the occurrence of seizures and thus promote the development of drug-drug interactions (DDIs). Therefore, their co-administration with lacosamide may affect their pharmacokinetic and pharmacological effects. In a clinical study, when lacosamide was used in combination with an enzyme inducer of anticonvulsants such as carbamazepine, phenobarbital, or phenytoin, the concentration of lacosamide was lower than when used alone (Markoula et al., 2014). Lacosamide is metabolized by several cytochrome P450 (CYP) enzymes (CYP2C19, CYP2C9, and CYP3A4) (Cawello et al., 2014; Ahn et al., 2022), the main metabolite of which is O-Desmethyl-lacosamide (ODL) (Figure 1B). The first-generation anti-epileptic drug carbamazepine induces CYP3A4, CYP1A2, CYP2C19, so carbamazepine can alter the pharmacokinetic profile of lacosamide (Lakehal et al., 2002). Lacosamide can cause arrhythmias, and patients with diabetic peripheral neuropathy and cardiovascular disease are prone to atrial arrhythmias (DeGiorgio et al., 2013; Malissin et al., 2013). Digoxin is primarily used to treat heart failure and atrial fibrillation because it has a limited range of therapeutic targets. It is used in combination with lacosamide, and has no significant effect on the absorption of lacosamide and the elimination of metabolites (Cawello and Bonn, 2012).

In addition, as global life expectancy increases, the number of elderly epilepsy patients is increasing. Elderly patients with epilepsy are at great risk of cardiovascular disease, and many epilepsy patients with cardiovascular and cerebrovascular diseases in the treatment of the primary disease at the same time to take cardiovascular drugs to control the disease (Husein et al., 2021). Due to the chronic nature of epilepsy, most patients need to take anti-epileptic drugs for a long period of time, so it is more important to take into account drug interactions in order to alleviate the patient’s condition (Cawello, 2015). In order to improve the effectiveness and safety of clinical drugs, a rapid and accurate method for the determination of drug concentration is needed. The main methods reported for the determination of lacosamide included gas chromatography-mass spectrometer (GC-MS) (Nikolaou et al., 2015; Mouskeftara et al., 2020), high performance liquid chromatography (HPLC) (Kestelyn et al., 2011; Shah et al., 2012), and liquid chromatography tandem mass spectrometry (LC-MS/MS) (Kim et al., 2012; Payto et al., 2014; Korman et al., 2015; Bharwad et al., 2020; Qiu et al., 2022). The significant features of these methods and the detection methods in this experiment are compared in Table 1.

**TABLE 1:** Summary of methods for the measurement of lacosamide in the literatures.

Sample preparation	Metabolite	IS	Run time (min)	Methodology	LLOQ (μg/mL)	HLOQ (μg/mL)	Ref
Rat plasma (100 μL) plus IS (10 μL) protein precipitation with acetonitrile	O-desmethyl lacosamide	lamotrigine	2.0	UPLC-MS/MS	0.002	10	Our method
Human plasma (25 μL) plus methanol precipitated solution (150 μL) containing IS (5 μg/mL)	O-desmethyl lacosamide	lacosamide-13C	6.5	LC–MS/MS	0.95	30.29	Payto et al. (2014)
Human plasma (500 μL) plus IS (25 μL) extracted with ethyl acetate	None	moclobemide	7.0	GC-MS	2	100	Mouskeftara et al. (2020)
Human plasma (200 μL) plus IS (20 μL) derivatized by silylation	None	levetiracetam-d6	11.5	GC-MS	0.2	20	Nikolaou et al. (2015)
Human plasma (100 μL) plus IS (25 μL) protein precipitation with methanol	None	AC-Phe-Nhme	25	HPLC-UV	0.5	12.5	Kestelyn et al. (2011)
Rat plasma (200 μL) plus IS (10 μL) extracted with ethyl acetate	None	nevirapine	17	HPLC	0.025	10	Shah et al. (2012)
Human plasma (50 μL) plus methanol precipitated solution (200 μL) containing IS (1 μg/mL)	None	lacosamide-13C	0.2	SPE-MS/MS	0.5	50	Korman et al. (2015)
Rat plasma (50 μL) plus IS (100 μL) protein precipitation with acetonitrile	None	LCD001	4.5	LC-MS/MS	0.0003	1	Kim et al. (2012)
Rat plasma (50 μL) plus acetonitrile precipitated solution (150 μL) containing IS (50 ng/mL)	None	midazolam	6.5	UPLC-MS/MS	0.5	5	Qiu et al. (2022)
Human plasma (150 μL) plus IS (50 μL) protein precipitation with methanol and acetonitrile	None	lacosamide-13C	2.2	UPLC-MS/MS	0.02	20	Bharwad et al. (2020)

However, to the best of our knowledge, only one analytical method for the simultaneous measurement of lacosamide and its metabolite ODL in biological matrices by LC-MS/MS has been reported. In this investigation, the LC-MS/MS method for the measurement of lacosamide and ODL had long analysis run time (6.5 min) and low sensitivity (0.95 μg/mL) (Payto et al., 2014). Thus, in this study, we firstly established a simple, quick and sensitive ultra performance liquid chromatography tandem mass spectrometry (UPLC-MS/MS) method for the measurement of the concentrations of lacosamide and ODL in plasma and rat liver microsomes (RLM) using lamotrigine as internal standard (IS). In addition, the effect of nisoldipine on lacosamide in RLM and its mechanism were investigated. Moreover, since our *in vitro* results suggested that nisoldipine had a significant inhibitory effect on lacosamide in RLM, we performed *in vivo* drug interaction experiments in the presence or absence of nisoldipine in rats, and evaluated the changes of pharmacokinetic parameters of lacosamide and ODL.

## Materials and methods

### Chemicals and reagents

Lacosamide (purity, 98%), lamotrigine (purity, 98%, used as internal standard, IS), and ODL, were supplied from Beijing Sunflower Technology Development Co., Ltd (Beijing, China). Nisoldipine (purity, 99%), Lercanidipine (purity, 99%), Nitrendipine (purity, 98%), Nimodipine (purity, 98%), Lacidipine (purity, 98%), Felodipine (purity, 98%) and Nicardipine (purity, 98%) were provided by Shanghai Canspec Scientific Instruments Co., Ltd. (Shanghai, China). Acetonitrile (HPLC grade) and methanol (HPLC grade), were produced by Merck Company (Darmstadt, Germany). Ultra-pure water was prepared by a Milli-Q water purification system manufactured by Milli-Q (Millipore, Bedford, USA). All other chemicals and biologicals were of analytical grade or above.

### Liquid chromatographic and mass spectrometric conditions

A Waters UPLC-MS/MS System comprised of a Waters Xevo TQ-S triple quadrupole tandem mass spectrometer (Milford, MA, USA) and a Waters Acquity UPLC I-Class system (Milford, MA, USA) was used for chromatographic analysis using an Acquity BEH C18 chromatography column (2.1 mm × 50 mm, 1.7 μm) at a flow rate of 0.40 mL/min. In addition, other conditions were set as follows: injection volume of 6.0 μL, autosampler temperature of 10°C, column temperature of 40°C. 0.1% formic acid in water (solution A) and acetonitrile (solution B) was used to form the mobile phase with a gradient elution as below: 0–0.5 min, 90% A; 0.5–1.0 min 90%–10% A; 1.0–1.4 min, 10% A; and 1.4–1.5 min, 10%–90% A. 90% A was then maintained at 1.5–2.0 min to reach equilibrium. The entire run time was 2.0 min.

A Waters Xevo TQ-S triple quadrupole tandem mass spectrometer was combined with electrospray ionization (ESI) in positive ion mode for mass spectrometry. Measurements were conducted by multiple reaction monitoring (MRM). The quantitative ion pairs and related parameters of lacosamide, its metabolite ODL, and IS are listed in Table 2. The Masslynx 4.1 software (Waters Corp, Milford, MA, USA) was used to collect, process, and control the apparatus.

**TABLE 2:** The Quantitative Ion Pairs and Related Parameters of lacosamide, Its Metabolite ODL, and IS.

Compound	Parent (*m/z*)	Daughter (*m/z*)	Cone (V)	Collision (eV)
Lacosamide	251.1	108.2	20	25
ODL	237.1	108.2	20	10
IS	256.0	145.0	10	20

### Rat liver microsomes

Six blank rat livers were weighed and homogenized with 0.01 mM cold PBS buffer containing 0.25 mM sucrose solution (stored at 4°C). For 15 min, the homogenate was centrifuged at 11,000 rpm. After that, discard the precipitation, and the supernatant was transferred to a fresh tube and centrifuged once again for 15 min at 11,000 rpm. Supernatant was then spun at 100,000 ×*g* for 1 h. Finally, the supernatant was discarded and the microsomal microspheres were frozen in a 0.01 mM PBS suspension and kept at −80°C. The Bradford Protein Assay Kit (Thermo Scientific, Waltham, MA, USA) was used to measure the protein concentration (Wang et al., 2015).

### Preparation of standard curve and quality control (QC) samples

A certain amount of lacosamide was accurately weighed, and diluent (methanol) was added into the EP tube to dissolve. After shaking, 1.00 mg/mL lacosamide reserve solution was obtained. A series of standard working solutions with concentration gradients of 20, 100, 500, 1,000, 5,000, 10,000, 50,000 and 100,000 ng/mL were obtained by dilution with methanol. According to the same method, ODL were prepared and a series of concentration gradients were obtained: 10, 20, 50, 100, 500, 1,000, 5,000 and 10,000 ng/mL. From the prepared standard working solution, 10 μL lacosamide and 10 μL ODL were added to 80 μL blank matrix to prepare calibration standards with concentrations of 2, 10, 50, 100, 500, 1,000, 5,000 and 10,000 ng/mL for lacosamide, and 1, 2, 5, 10, 50, 100, 500 and 1,000 ng/mL for ODL, and these standard curves were determined. Lower limit of quantification (LLOQ) and three Quality Control (QC) samples were obtained by the same method. The LLOQ of lacosamide and ODL were 2 ng/mL and 1 ng/mL, respectively, and the three QC samples of lacosamide and ODL were 5, 4,000 and 8,000 ng/mL, and 2, 400 and 800 ng/mL, respectively. All chemicals and solutions were stored at - 80°C for further use.

Calibration curves (80–40000 ng/mL) in RLM were established by preparing calibration products according to the above method. The three QC concentrations were 200, 16,000 and 32,000 ng/mL, respectively, and the LLOQ was 80 ng/mL.

### Pre-treatment of samples

Protein precipitation was used to remove proteins from plasma and extract the test materials. 100 μL plasma sample was mixed with 10 μL IS working solution (1 μg/mL) and 300 μL acetonitrile, and centrifuged at 13,000 rpm for 10 min at 4 °C. The supernatant was obtained and aspirated into a sample bottle. Finally, 6.0 μL of sample was injected for analysis.

### Method validation

The parameters of this test, which was conducted according to with the FDA bioassay, included selectivity, calibration curve, LLOQ, accuracy and precision, matrix effect, recovery, and stability (US Food and Drug Administration, 2018).

Different batches of matrix-match samples were treated to examine the selectivity of the UPLC-MS/MS method. Analysis of blank matrix samples (neither analyte nor IS), and real sample is done to determine whether endogenous chemicals interfere with the retentions of the analytes and IS.

The concentrations of lacosamide and ODL in matrix were taken as the horizontal coordinate, and the ratio of the two to the IS peak area was taken as the vertical coordinate. The weighted least square method was used to perform regression operations, and the calibration curves of lacosamide and ODL were obtained respectively. LLOQ is the minimum concentration of the calibration curve and should be accurate and precise in the range of ±20%.

To achieve inter-day and intra-day precision and accuracy, QC samples were analyzed in five batches over three consecutive days. Separate calibration is performed on each verification day. Relative standard deviation (RSD%) is used to indicate accuracy, and relative error (RE%) is used to indicate accuracy. Accuracy of ±15% or less, precision of 15% or less is considered acceptable.

Lacosamide and ODL standard solutions were added to the treated various blank matrices to prepare three QC concentrations. The peak areas obtained were compared with those obtained from three QC levels of pure standard solutions, that is, matrix effect. The recovery was obtained by comparing the peak area before extraction of lacosamide and ODL with the peak area after extraction at the three QC levels.

Three concentrations of QC matrix-match samples were placed in various conditions to test the stability of lacosamide and ODL for short-term, long-term, three freeze-thaw cycles, and at autosampler. Sample storage at room temperature for at least 3 h was used to test the short-term stability. After 21 days of storage at −80°C, samples were tested to establish their long-term stability. By assessing the stability of three freeze-thaw cycles from freezing (−80°C) to thawing (room temperature) three times, the stability of the samples was assessed. Additionally, the stability of the produced samples was assessed after 4 h of storage in an autosampler at 10°C.

### Enzyme reaction of lacosamide using RLM

The range of 20–2000 μM of lacosamide had been dissolved in DMSO to comply with the K_m_ (Michaelis-Menten constant) sequence concentrations. Incubation system of 200 μL was made up of 100 mM of PBS buffer, 0.2 mg/mL of RLM, 1 mM of NADPH, and 20–2000 μM of lacosamide. In addition to NADPH, the solution was mixed and pre-incubated at 37°C for 5 min prior to the addition of 1 mM NADPH, followed by incubation for 50 min and placed at - 80°C to discontinue the reaction. After complete completion of the enzyme reaction, the mixture was added 10 μL of 200 ng/mL IS solution and 300 μL of acetonitrile (protein precipitant). The mixture was then swirled for 2 min and centrifuged at 13,000 rpm for an additional 10 min, and the supernatant of 100 μL was placed in an injector for the quantification by UPLC-MS/MS.

### Effect of cardiovascular drugs on the metabolism of lacosamide in RLM

In the RLM incubation system, the K_m_ of lacosamide was 1,016 μM. In order to investigate the potential DDIs of lacosamide, the 200 μL system remained unchanged, and using K_m_ as the concentration of lacosamide in the RLM system, 7 cardiovascular drugs (nitrendipine, lercanidipine, nisoldipine, lacidipine, felodipine, nimodipine, nicardipine, 100 μM of each drug as the inhibitor) were tested for inhibition on the metabolism of lacosamide. The following reaction procedure was identical to the above-mentioned enzyme reaction. The experiment had to be redone with an inhibition rate of 80% or higher.

The half maximal inhibitory concentration (IC_50_) of inhibitors (nisoldipine, lacidipine, felodipine, nimodipine, nicardipine) against lacosamide was determined in RLM. The cardiovascular drugs was dissolved in DMSO and diluted with DMSO at a concentration gradient of the IC_50_ was 0, 0.01, 0.1, 1, 10, 25, 50 and 100 μM, and the concentration of lacosamide was 1,016 μM in RLM. The subsequent processing steps were the same as above to obtain the IC_50_ values of inhibitors in RLM. To identify the mechanisms underlying the inhibitory effect of nisoldipine on lacosamide, 0, 1.7, 3.4 and 6.8 μM of nisoldipine (based on the IC_50_ value), and 254, 508, 1,016, and 2032 μM of lacosamide (according to the K_m_ value) in the RLM system, were selected. The subsequent reaction and treatment process were the same as the described above.

### *In vivo* pharmacokinetic study

18 Sprague-Dawley (SD) male rats (200 ± 10 g) were bought from the Animal Experimental Center of the First Affiliated Hospital of Wenzhou Medical University and were randomly assigned to three groups (*n* = 6): group A (control group) and group B, C (experimental group). The animals fasted for 12 h before the experiment, with no restricted water intake. Lacosamide, felodipine, and nisoldipine were prepared with suspension of 0.5% sodium carboxymethyl cellulose (CMC-Na) solution and administered orally. Groups B and C were given felodipine (1 mg/kg), and nisoldipine (1 mg/kg) by gavage, respectively, and the same amount of CMC-Na solution was administered to group A. After 30 min, all rats were received 10 mg/kg of lacosamide by gavage. Tail venous blood was collected at 0.333, 0.667, 1, 1.5, 2, 3, 4, 6, 8, 12, 24 and 48 h after dosing. The blood sample (0.3 mL) was centrifuged at 8,000 rpm for 5 min 100 μL of plasma was obtained and frozen at - 80°C for further processing. The subsequent treatment was carried out according to the chapter of Pre-treatment of samples.

### Data analysis

The GraphPad Prism 9.0 software was used to generate the K_m_, IC_50_, the Lineweaver-Burk plot, and the mean plasma concentration-time curve. Drug and Statistics (DAS) software (version 3.0 software, Mathematical Pharmacology Professional Committee of China, Shanghai, China) with non-compartment model analyses was used to obtain the pharmacokinetic parameters of lacosamide and ODL in rats. The comparison of the pharmacokinetic parameters among three groups in rats was performed with SPSS (version 26.0; SPSS Inc., Chicago, IL, USA), with one-way ANOVA, and the *p*-value <0.05 was regarded as statistically significant.

## Results

### Method validation

**FIGURE 2:**
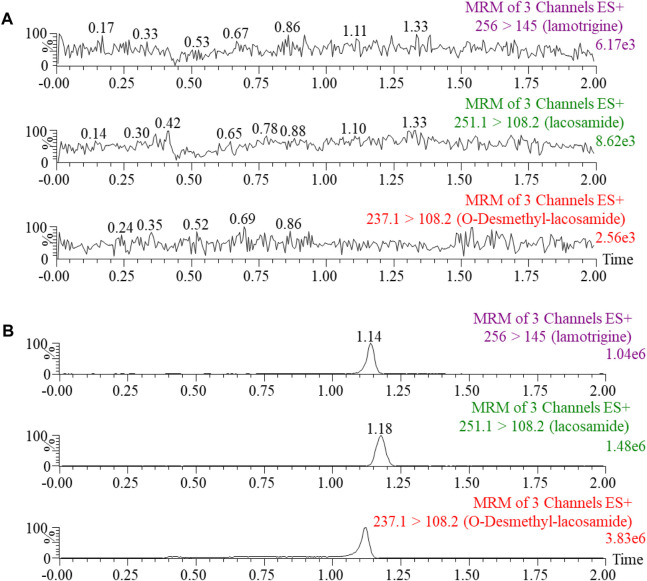
Blank rat plasma samples **(A)**: no analyte, no IS). Typical MRM chromatograms of lacosamide, O-Desmethyl-lacosamide and lamotrigine (IS) in rat plasma **(B)**.

The method for the quantification of lacosamide and ODL had been successfully established, as illustrated in Figure 2, with no interference peaks. In this study, the regression equation of calibration standard curve was constructed as follows: lacosamide, y= (0.000764*x + 0.001259, *r* ^2^ = 0.996); ODL in plasma, y= (0.005327*x + 0.003107, *r* ^2^ = 0.999); ODL in RLM, y= (0.004099*x + 0.000403, *r* ^2^ = 0.998). The calibration standard curves illustrated excellent linearity at a concentration range of 2–10000 ng/mL for lacosamide and that of 1–1,000 ng/mL for ODL in plasma, also 80–40000 ng/mL for ODL in RLM.

Four concentration levels of each analyte have been employed to measure the precision and accuracy. Lacosamide and ODL have intra-day and inter-day precision of less than 15% and accuracy of ±15% at three QC levels. As for LLOQ, the precision was below 20% and the accuracy was within ±20%. All the results in various matrices are presented in Table 3.

**TABLE 3:** Evaluation of the Intra-Day and Inter-Day Precision and Accuracy by the Proposed UPLC-MS/MS Method for Determination of lacosamide and ODL in various matrices (n = 5).

Matrix	Analytes	Concentration (ng/mL)	Intra-Day		Inter-Day	
			Precision (RSD %)	Accuracy (RE %)	Precision (RSD %)	Accuracy (RE %)
Plasma	Lacosamide	2	19.9	−3.5	18.7	7.6
		5	2.4	−1.0	3.2	2.5
		4,000	4.8	11.2	3.6	11.5
		8,000	3.3	−3.4	2.9	3.6
	ODL	1	10.7	−0.9	8.9	3.1
		2	9.2	2.0	6.9	0.1
		400	2.6	2.0	3.7	0.7
		800	6.4	5.5	5.3	5.0
RLM	ODL	80	12.3	0.0	11.6	3.0
		200	4.5	3.5	6.0	5.0
		16,000	3.7	5.5	7.6	11.8
		32,000	6.7	2.5	6.4	4.3

Three QC levels were used to study the recovery and matrix effects. As shown in Table 4, the recovery rates of lacosamide and ODL in rat plasma ranged from 99.0% to 110.4% and 89.9%–102.3%, respectively. The matrix effect of lacosamide and ODL were calculated to be within the acceptable range (89.8%–111.6% and 95.7%–112.5%). The recovery and matrix effect in RLM also met the requirements of the guidelines on bioanalytical method validation. Thus, matrix effect had little influence on the ionization of the analytes, nor did it influence the precision of UPLC-MS/MS optimization.

**TABLE 4:** Matrix effect and recovery rate of lacosamide and ODL in various matrices (n = 5).

Matrix	Analytes	Concentration (ng/mL)	Recovery rate (%)		Matrix effect (%)	
			Mean ± SD	RSD%	Mean ± SD	RSD%
Plasma	Lacosamide	5	104.8 ± 12.9	12.3	111.6 ± 4.5	4.0
		4,000	110.4 ± 3.7	3.3	89.8 ± 5.8	6.5
		8,000	99.0 ± 7.9	8.0	92.8 ± 7.7	8.3
	ODL	2	92.5 ± 4.6	5.0	96.0 ± 3.6	3.8
		400	89.9 ± 5.0	5.6	95.7 ± 6.8	7.1
		800	102.3 ± 3.6	3.6	112.5 ± 2.6	2.3
RLM	ODL	200	102.8 ± 5.6	5.4	99.6 ± 11.8	11.9
		16,000	99.4 ± 4.6	4.6	109.2 ± 6.9	6.3
		32,000	104.9 ± 8.2	7.8	105.3 ± 4.3	4.1

The stability of lacosamide and ODL in various matrices was determined by the stability test, and were expressed in Table 5. The results showed that lacosamide and ODL in matrix-match samples were stable after analysis under four different conditions: room temperature for 3 h, 10°C for 4 h, three complete freezing (−80°C)/thawing (RT) cycles, or storage at −80°C for 21 days. The stability test results were in the range of error (±15%).

**TABLE 5:** Stability Results of lacosamide and ODL in various matrices Under Different Conditions (n = 5).

Matrix	Analytes	Concentration (ng/mL)	Room temperature (3 h)		10°C (4 h)		Three freeze-thaw		21 days	
			RSD (%)	RE (%)	RSD (%)	RE (%)	RSD (%)	RE (%)	RSD (%)	RE (%)
Plasma	Lacosamide	5	2.4	10.0	9.5	−1.7	4.0	7.3	12.7	1.0
		4,000	3.9	5.8	2.6	12.7	3.4	1.5	1.0	−4.6
		8,000	0.7	−10.4	4.7	−1.9	2.2	−14.4	1.5	−2.4
	ODL	2	3.4	4.4	5.8	−0.3	12.3	−1.6	5.0	9.3
		400	4.1	0.0	2.3	−7.4	4.6	1.6	2.5	−5.1
		800	4.4	0.6	2.8	−7.4	2.9	4.1	5.8	0.0
RLM	ODL	200	9.5	−4.3	5.2	9.9	4.8	−10.4	10.9	−9.7
		16,000	2.9	−0.2	3.2	10.4	2.8	−0.9	3.5	1.1
		32,000	1.6	1.9	7.3	2.6	3.4	−1.0	2.2	3.9

### Screening drugs with potential interactions in combination with lacosamide

**FIGURE 3:**
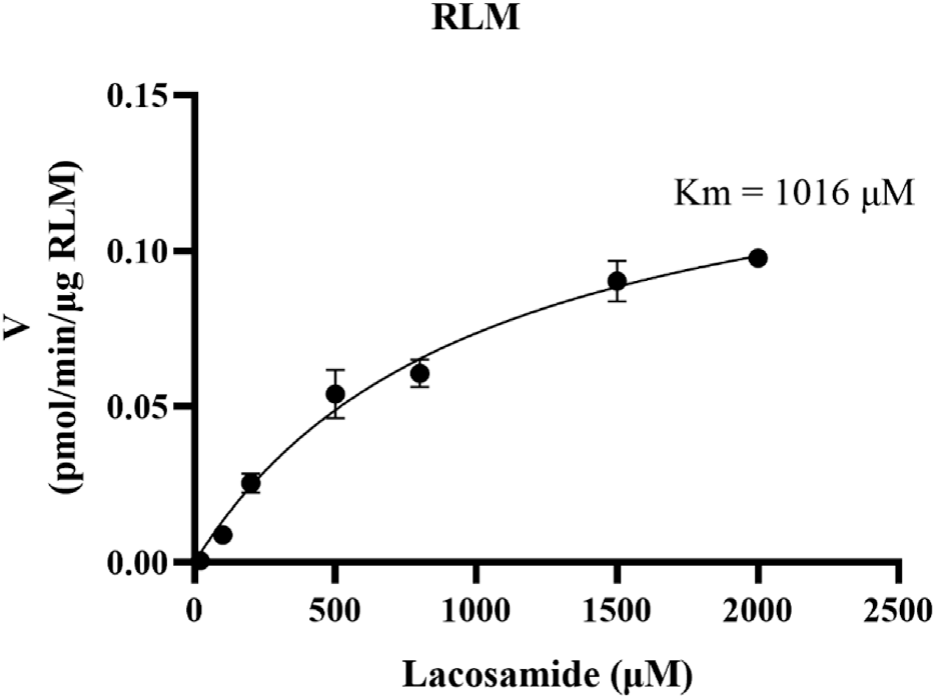
Michaelis–Menten kinetics of lacosamide in RLM.

**FIGURE 4:**
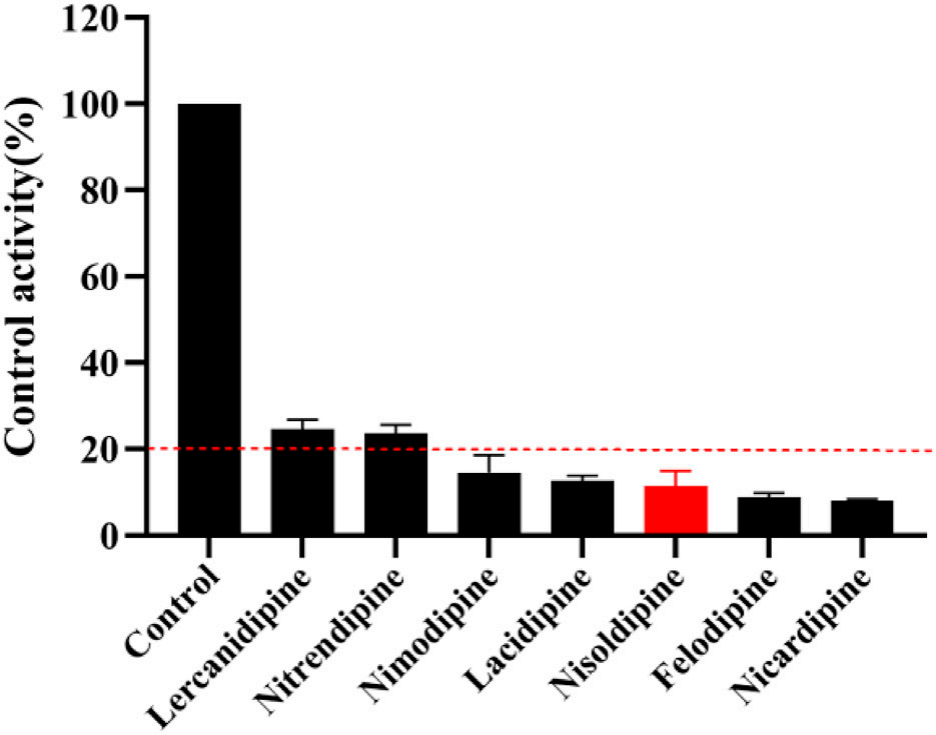
Comparison of the inhibitory effects of 7 cardiovascular drugs on lacosamide in RLM.

The K_m_ value for lacosamide was determined by non-linear regression of the rate of reaction to the concentration of the substrate (Figure 3). The K_m_ values of lacosamide was 1,016 μM in RLM. In this study, 7 cardiovascular drugs that may be used in combination with lacosamide were selected, and it was found that 5 drugs (nisoldipine, felodipine, lacidipine, nimodipine, and nicardipine) with inhibition rates greater than 80% were screened out (Figure 4).

### Effect of nisoldipine on the enzyme kinetics of lacosamide *in vitro*

**FIGURE 5:**
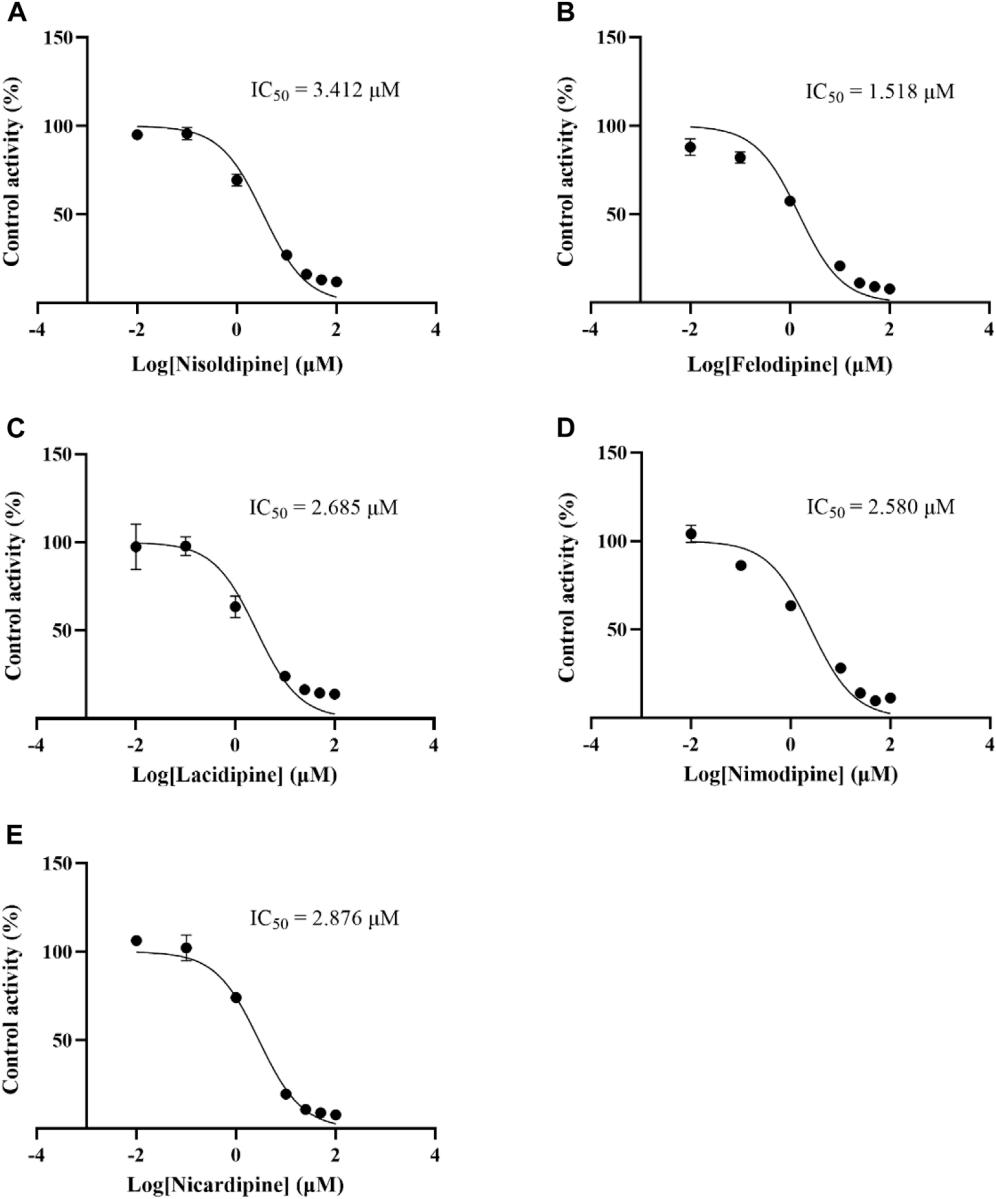
IC_50_ curves of 5 cardiovascular drugs on lacosamide metabolism in RLM. Nisoldipine **(A)**, Felodipine **(B)**, Lacidipine **(C)**, Nimodipine **(D)**, Nicardipine **(E)**.

**FIGURE 6:**
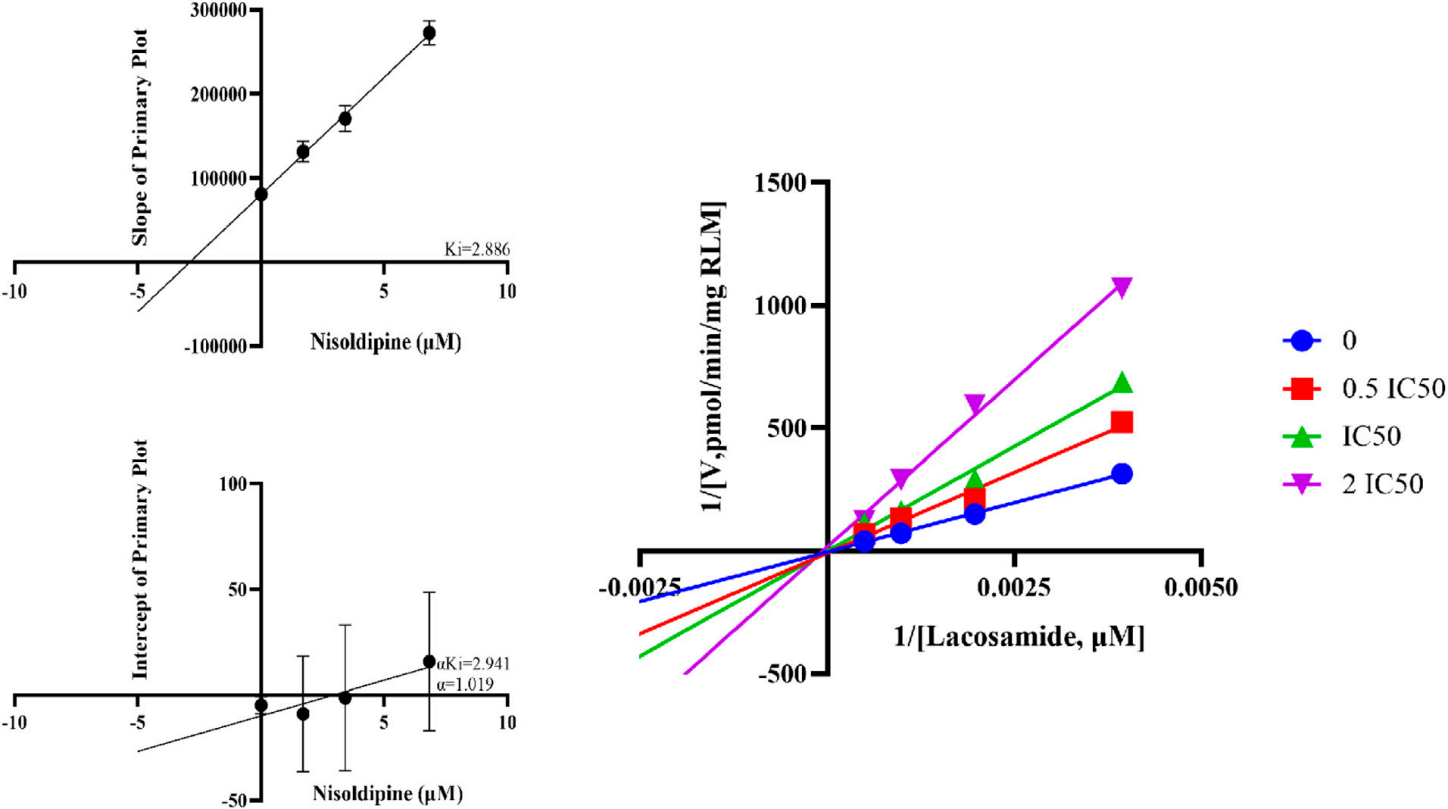
Linewever-burk plot, secondary diagram of Ki and secondary diagram of αKi inhibiting lacosamide metabolism at different concentrations of nisoldipine in RLM.

The IC_50_ curves of 5 cardiovascular drugs on lacosamide metabolism in RLM are displayed in Figure 5. The assessment of inhibition revealed that nisoldipine, felodipine, lacidipine, nimodipine, and nicardipine could significantly decrease the concentration of ODL (IC_50_ were 3.412, 1.518, 2.685, 2.580 and 2.876 μM, respectively). The IC_50_ was below 10 μM, suggesting a modest inhibition of the metabolism of lacosamide *in vitro*. The inhibitory mechanisms of nisoldipine on lacosamide in RLM were further investigated, and it inhibited the metabolism of lacosamide with a Ki of 2.886 and αKi of 2.941, by a mixed inhibition mechanism, involving un-competitive as well as non-competitive inhibition, in accordance with the Lineweaver-Burk plots (Figure 6).

### Effect of nisoldipine on the pharmacokinetics of lacosamide *in vivo*

**FIGURE 7:**
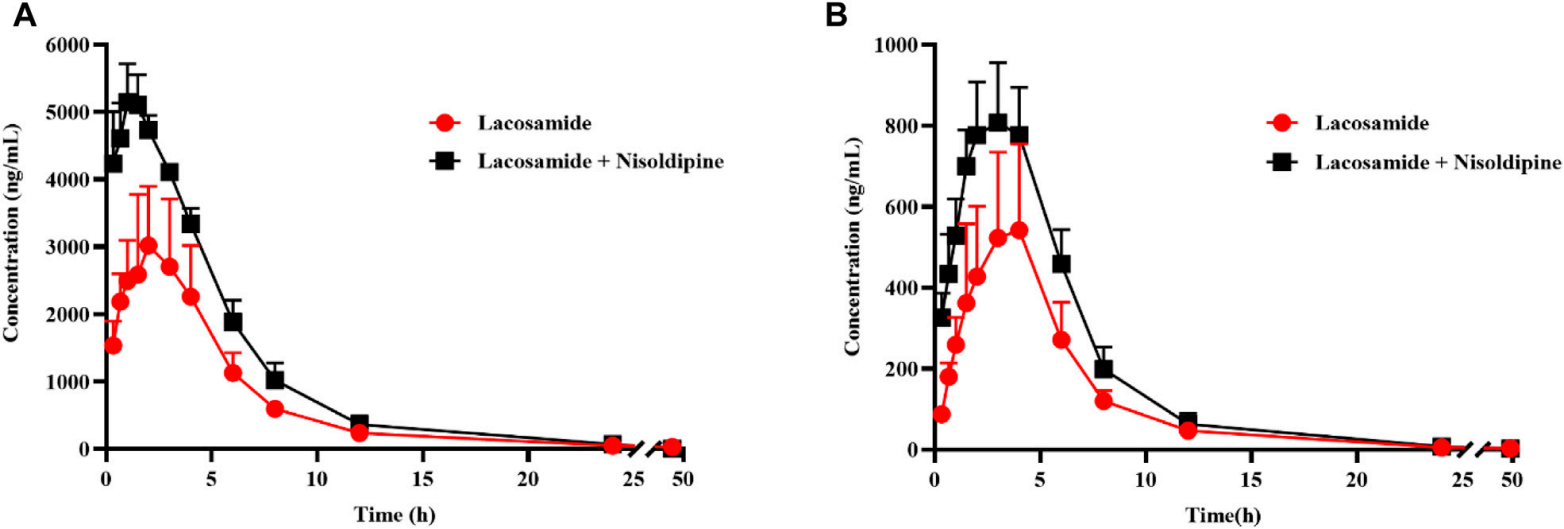
Mean concentration-time curve of lacosamide **(A)**, and ODL **(B)** in rats. Data are presented as the means ± SD, n = 6.

The average concentration-time curves of lacosamide and ODL in rats are shown in Figure 7; Tables 6, 7 show the main pharmacokinetic results obtained from non-compartment model analyses in rats with or without nisoldipine. The results indicated that the combination of nisoldipine and lacosamide was the most effective. The AUC_(0-t)_, AUC_(0-∞)_ and C_max_ of lacosamide were significantly increased when nisoldipine was administered in combination with lacosamide in rats, while CL_z/F_, T_max_, and, V_z/F_ were significantly decreased. And, there was no significant change in MRT_(0-t)_, MRT_(0-∞)_ and t_1/2z_. These results suggested that nisoldipine could increase the plasma exposure of lacosamide in SD rats, demonstrating that nisoldipine might be a potential interaction drug with lacosamide. With respect to ODL, the AUC_(0-t)_, AUC_(0-∞)_ and C_max_ increased significantly, the MRT_(0-t)_, MRT_(0-∞)_, CL_z/F_ and V_z/F_ decreased significantly, and t_1/2z_ and T_max_ had no significant difference when nisoldipine was administered. As for felodipine, it did not change the exposure of lacosamide and ODL in rats.

**TABLE 6:** The Pharmacokinetic Parameters of Lacosamide in the Presence and Absence of nisoldipine (n = 6).

Parameters	Lacosamide	Lacosamide + nisoldipine
AUC_(0-t)_ (ng/mL*h)	18,734.31 ± 4,192.44	31,364.27 ± 3251.79***
AUC_(0-∞)_ (ng/mL*h)	18,742.60 ± 4,200.86	31,367.45 ± 3255.93***
MRT_(0-t)_ (h)	6.54 ± 2.31	5.07 ± 0.92
MRT_(0-∞)_ (h)	7.01 ± 3.14	5.11 ± 0.92
t_1/2z_ (h)	3.49 ± 1.03	3.26 ± 0.71
T_max_ (h)	2.08 ± 0.49	1.42 ± 0.38*
CL_z/F_ (L/h/kg)	0.60 ± 0.13	0.32 ± 0.00**
V_z/F_ (L/kg)	2.72 ± 0.58	1.49 ± 0.19***
C_max_ (ng/mL)	3164.24 ± 1,070.20	5,297.66 ± 466.35***

AUC, area under the plasma concentration-time curve, t_1/2z_ elimination half time, T_max_ peak time, CL_z/F_ plasma clearance, C_max_ maximum blood concentration *p < 0.05, **p < 0.01, ***p < 0.001, compared with the group A.

**TABLE 7:** The Pharmacokinetic Parameters of ODL in the Presence and Absence of nisoldipine (n = 6).

Parameters	Lacosamide	Lacosamide + nisoldipine
AUC_(0-t)_ (ng/mL*h)	3422.11 ± 1,059.19	5,482.80 ± 807.91**
AUC_(0-∞)_ (ng/mL*h)	3423.38 ± 1,061.55	5,495.81 ± 817.99**
MRT_(0-t)_ (h)	6.01 ± 0.64	5.30 ± 0.39*
MRT_(0-∞)_ (h)	6.28 ± 0.74	5.44 ± 0.40*
t_1/2z_ (h)	3.26 ± 0.54	3.11 ± 0.21
T_max_ (h)	3.50 ± 0.55	2.83 ± 0.75
CL_z/F_ (L/h/kg)	3.11 ± 0.77	1.86 ± 0.32**
V_z/F_ (L/kg)	14.99 ± 5.04	8.38 ± 1.71*
C_max_ (ng/mL)	558.05 ± 220.49	829.00 ± 128.67*

AUC, area under the plasma concentration-time curve, t_1/2z_ elimination half time, T_max_ peak time, CL_z/F_ plasma clearance, C_max_ maximum blood concentration *p < 0.05, **p < 0.01, compared with the group A.

## Discussion

People with epilepsy often have complications or underlying conditions that may be combined with cardiovascular medications (Cawello, 2015). Depending on the drug’s dose as well as its specific metabolic path, DDIs may result in negative reactions to drugs. Lacosamide is metabolized by multiple cytochrome P450 enzymes, including CYP3A4, CYP2C9, and CYP2C19, producing the primary inactive O-demethylated metabolite.

To better analyze lacosamide, ODL, and IS and produce more precise analytical results, the MS settings were improved in this experiment. Lacosamide, ODL, and IS have good peak and separation effects following the optimized gradient elution process with 0.1% formic acid in water and acetonitrile as mobile phase, and the retention time is 1.18 min, 1.12 min and 1.14 min, respectively. The quantitative fragment ions of lacosamide, ODL and IS were *m/z* 251.1→108.2,*m/z* 237.1→108.2 and *m/z* 256.0→145.0, respectively. Samples need to be processed before UPLC-MS/MS detection, and we used acetonitrile one-step precipitation extraction to reduce interference from endogenous substances, especially proteins, and to obtain permitted recovery and matrix effects.

Many cardiovascular drugs are substrates for CYP3A4, CYP2C19, and CYP2C9, or inhibitors of these enzymes (Yuan et al., 2014; Zhou et al., 2014; Fuhr et al., 2022). In this study, after screening 7 potential cardiovascular drugs before the *in vivo* study, 5 drugs with higher inhibitory rates were selected for their IC_50_ values, all of which were less than 10 μM, and 2 drugs (felodipine, and nisoldipine) were selected for *in vivo* pharmacokinetic experiments in rats. All two cardiovascular drugs are calcium channel blockers that are widely used in the treatment of cardiovascular disease and are clinically approved for the treatment of hypertension (Wei et al., 2012; Wu et al., 2015).

As far as we know, nisoldipine is a substrate of CYP3A4, but its inhibitory effect on CYP450 has not been reported. In this study, we firstly studied the inhibitory mechanism of nisoldipine on lacosamide in RLM through the production of ODL *in vitro*. The results showed that nisoldipine could significantly inhibit the formation of ODL and showed moderate inhibition on lacosamide, and its inhibitory effect occurred through a mixed inhibitory mechanism (non-competitive inhibition and un-competitive inhibition). These results suggested that nisoldipine may exhibit mixed inhibition through multiple pathways.

Previously reported t_1/2z_, CL_z/F_ and V_z/F_ of lacosamide alone in rats were similar to those in our study, with slight differences in C_max_ and T_max_, such differences may have resulted from individual variances between rats. The values of AUC_(0-t)_ and MRT_(0-t)_ are roughly half of those in this study, which are 9270.3 ± 3231.7 ng/mL*h and 3.6 ± 0.5 h respectively (Qiu et al., 2022), while the values of these two parameters in this experiment are 18,734.3 ± 4,192.4 ng/mL*h and 6.54 ± 2.31 h respectively. The reason may be that the oral dose in the literature (5 mg/kg) is half of the dose in this experiment (10 mg/kg). The pharmacokinetic parameters of ODL in rats have not been reported despite in healthy subjects, where the previously reported value of C_max_ (0.459 μg/mL) in healthy subjects was consistent with our study of C_max_ (558.0 ± 220.5 ng/mL) (Cawello et al., 2014).

The results of pharmacokinetic experiments in rats showed that in the present of nisoldipine, AUC and C_max_ were increased significantly, and CL_z/F_ were decreased by about 50%, so the metabolism of lacosamide were slowed down and/or drug absorption were increased. Nisoldipine had no significant effect on the final t_1/2z_ of lacosamide, indicating that nisoldipine had a moderate effect on lacosamide clearance, and the effect on the final t_1/2z_ was not as significant as that on AUC. The AUC, C_max_, and CL_z/F_ values for ODL were consistent with the prototype in the presence of nisoldipine, according to the pharmacokinetic data of the metabolite. This result could be explained by lacosamide’s higher bioavailability in the presence of nisoldipine, which suggests that greater amounts of lacosamide has the potential to be transformed into ODL. However, *in vivo* studies in rats cannot properly predict the situation in people since the role of the CYP450 enzymes for substrate metabolism in humans and rats differ, and additional research is required to confirm this in clinical investigations.

## Conclusion

All in all, this study assessed the impact of DDIs on the metabolism of lacosamide. Through sample pre-treatment and complete methodology verification, the method was characterized by simple operation, high recovery, high sensitivity and specificity. When co-administered with lacosamide in rats, nisoldipine could significantly alter lacosamide and ODL pharmacokinetic parameters. In addition, we found that the inhibition of nisoldipine on lacosamide was consistent *in vivo* and *in vitro*. Due to the wide range of clinical applications of lacosamide and nisoldipine, we could provide basis for the appropriate and rational combination of lacosamide and nisoldipine, offer evidence for the joint development of antiepileptic drugs and cardiovascular drugs, and furnish support for personalized clinical medication.

## Data Availability

The raw data supporting the conclusion of this article will be made available by the authors, without undue reservation.
